# Therapeutic effect of transcranial direct current stimulation on neuropsychological symptoms of an elderly patient: A case report

**DOI:** 10.1590/1980-57642016dn11-030014

**Published:** 2017

**Authors:** Aline Iannone, Joaquim Brasil-Neto, Antonio Pedro Mello Cruz, Corina Satler, Nasser Allam

**Affiliations:** 1 Institute of Psychology, University of Brasília – Brasília, DF, Brazil.; 2 Dof Physiological Science, Institute of Biology, University of Brasília – Brasília, DF, Brazil.; 3 Faculty of Ceilândia, University of Brasília – Brasília, DF, Brazil.

**Keywords:** neuromodulation, tDCS, elderly patients, mild neurocognitive disorder, depression, chronic pain, neuromodulação, ETCC, Pacientes idosos, comprometimento cognitivo leve, depressão, dor crônica

## Abstract

Although growing evidence points to the potential therapeutic effects of transcranial Direct Current Stimulation (tDCS), there is still no consensus on the most appropriate protocol to be used in specific neurological and neuropsychological symptoms. This case report evaluated the neuromodulatory therapeutic effects of two 15-day courses of tDCS on an elderly female patient, aged 78 years with mild neurocognitive disorder, chronic pain and depression-related symptoms. Results indicated an overall significant improvement of cognitive and executive functions, as well as reduction in both depression and chronic pain symptoms. These results highlight the potential of tDCS as a safe and useful neuromodulatory clinical tool in the rehabilitation of elderly patients.

## RESUMO

Embora evidências crescentes apontem para os potenciais efeitos terapêuticos da estimulação transcraniana por corrente contínua (ETCC), ainda não há consenso sobre o protocolo mais adequado a ser usado em sintomas neurológicos e neuropsicológicos específicos. Este relato de caso avaliou os efeitos terapêuticos neuromoduladores de dois protocolos de 15 dias de ETCC em paciente idosa, com idade de 78 anos com comprometimento cognitivo leve, dor crônica e sintomas relacionados à depressão. Os resultados indicaram uma melhoria global significativa das funções cognitivas e executivas, bem como a redução tanto na depressão quanto nos sintomas da dor crônica. Isso destaca o potencial do ETCC como uma ferramenta clínica neuromoduladora segura e útil na reabilitação de pacientes idosos.

## INTRODUCTION

Extensive evidence points to the potential effects of transcranial direct current stimulation (tDCS) in ameliorating behavioral and cognitive symptoms in several neuropsychological and neuropsychiatric disorders including dementia,^1^^,^ 2 mild neurocognitive disorder,^3^ depression,^4^^,^ 5 chronic pain,^6^^,^ 7 as well as for improving cognitive function in healthy individuals.^8^^-^ 20

tDCS has become increasingly more accessible as a non-invasive therapeutic alternative in individual clinical settings. However, there is still no consensus about the most appropriate tDCS protocol to be used or on its comparative efficacy in special situations such as in elderly patients.^21^ The present case report explored the effects of two 15-day courses of tDCS on an elderly female patient with symptoms suggestive of mild neurocognitive disorder,^22^ depression and neuropathic pain.

## CASE REPORT

A 78-year-old female patient, with 12 years of education, graduated in Economics, returned to the NA Neuroscience Clinic, Brasilia-DF, Brazil, 7 months after an initial consultation in which she had been diagnosed as having mild neurocognitive disorder, depression, and chronic pain. The patient was accompanied by her adult daughter, who reported that her mother's symptoms had worsened over the past 7 months.

The patient was diagnosed with mild neurocognitive disorder, depression and neuropathic pain (fibromyalgia). Selective serotonin reuptake inhibitors were prescribed with no clear benefit. Diagnosis was based in anamnesis and scores derived from neuropsychological evaluations by a board-certified neuropsychologist using the following instruments and scales: [1] *overall cognition* was assessed by the Mini-Mental State Exam (MMSE); [2] *comprehension and reasoning* were evaluated by the Comprehension, Similarities and Vocabulary Tests (Wechsler Adult Intelligence Scale – WAIS-III); [3] *executive and attention* functions were assessed by the Digit Span (forward and backward), Arithmetic (WAIS-III), and Victoria Stroop Color-Word Test; [4] *episodic memory and short-term memory* were evaluated by the Rey Auditory Verbal Learning Test (RAVLT); [5] *verbal and visuoconstructive* abilities were assessed by the Rey-Osterrieth Complex Figure immediate recall Test (ROCFT); [6] *perceptual organization* was evaluated by the Block Design and Matrix Reasoning (WAIS-III); [7] *processing speed* was evaluated by the Digit Symbol (WAIS-III); [8] *verbal, phonemic and semantic fluency* were assessed by the Animal Categories, Fruit Categories and Letters FAS Tests; [9] *functionality* was scored by Pfeiffer's Functional Assessment Questionnaire (FAQ); and [10] *depression* was scored by the Beck Depression Inventory (BDI). Additionally, [11] *chronic neuropathic* pain was scored using a Visual Analog Scale (VAS), and a self-report scale pain assessment.

Given the lack of effect of other treatment modalities including selective serotonin reuptake inhibitors (Lexapro^®^ 20 mg), and anti-inflammatory and/or analgesic agents (Feldene^®^ 20 mg) in this case, together with the reported benefits of tDCS on executive function by several authors, compassionate use of tDCS was prescribed. A commercially available transcranial direct current stimulator (TransCranial^®^, Hong Kong, China) was used to deliver 2.0 mA for 30-min per session through saline-soaked sponge electrodes (surface 35 cm^2^). Electrode placement on the scalp was determined using the International 10-20 EEG system. The anode was positioned over the left prefrontal dorsolateral cortex (F3), and the cathode was positioned over the contralateral scalp (Fp2). Location and polarity of the electrodes, number of sessions, and current intensity were chosen on the basis of previously reported results demonstrating that anodal tDCS over the left prefrontal dorsolateral cortex (F3) for 30 min is capable of improving executive function.^9^ Because of the reported sciatic pain, a modified protocol was introduced after the third session: the active electrode (anode) was positioned over the primary motor cortex (M1) for 15 min and for the remaining 15 min the active electrode (anode) was positioned over the prefrontal dorsolateral cortex (F3). For both active electrodes, the return electrode (cathode) was placed over a contralateral scalp area (Fp2), and stimulation intensity was 2 mA. Many studies have shown that anodal tDCS over M1 or the dorsolateral prefrontal cortex (DLPFC) (2mA for 20 min on 5 consecutive days) has an analgesic effect after 3 weeks of treatment in patients with fibromyalgia.^23^

The patient underwent two courses of 15 daily tDCS sessions, with an interval of 7 months between the first (August 2014) and the second (March 2015) courses. During the first course, the patient was additionally submitted to cognitive training (CT), once a day, for 60 minutes, which was carried out on the same days, but not simultaneously with CT.

## RESULTS

tDCS sessions were very well tolerated by this elderly patient and may have played a role in the significant improvement on neuropsychological tests. After the first ten sessions of the first tDCS course, she also subjectively reported feelings of well-being and a decrease in chronic pain.

Table 1 illustrates comparisons between the patient's scores for each item immediately before the first tDCS course and after the second course.

**Table 1:** tDCS effects on different neuropsychological and neuropsychiatric parameters in the elderly patient before and after two tDCS clinical courses (August 2014 and March 2015). Each clinical course consisted of a 15-day tDCS protocol.

Instruments and measures			Before tDCS	After tDCS
Global cognition score	Mini-Mental State Exam (MMSE), n/26		26	29
Executive and attention functions	Digit Span Forward (WAIS), n/16		11	11
	Digit Span Backward (WAIS), n/14		07	08
	Digit Span (Forward - Backward), n/4		04	03
	Digit Span (Forward + Backward), n/30		17	18
	Arithmetic (WAIS), n/22		10	09
	Victoria Stroop Color-Word Test, (completion time)		65	50
Verbal fluency	Categories - Animals, n/15		13	20
	Categories - Fruits, n/15		08	10
	Letters - FAS, n/30		30	55
Comprehension and reasoning	Comprehension (WAIS), n/33		11	10
	Similarities (WAIS), n/38		12	15
	Vocabulary (WAIS), n/66		08	11
Episodic recall	Rey Auditory Verbal Learning Test (RAVLT)	Trial 1, n/15	06	06
		Trial 4 n/15	07	10
		Trial 5, n/15	08	09
		Trial B1, n/15 - Delayed Recall	06	02
		Trial A6, n/15	02	07
		Trial A7, n/15	03	04
		LOT, n/75 (A1-A5)	34	44
		Recognition	22	30
Nonverbal recall	Rey-Osterrieth Complex Figure immediate Recall Test (ROCFT)		32	34
Perceptual organization	Block design (WAIS), n/68		12	13
	Matrix reasoning (WAIS), n/26		15	13
Processing speed	Digit Symbol (WAIS), n/133		14	15
Visuoconstructive abilities	Rey-Osterrieth Complex Figure immediate Recall Test (ROCFT)		27	34
	Rey-Osterrieth Complex Figure delayed Recall		02	03
Neuropsychiatric symptoms and psychopathology	Beck Depression Inventory (BDI), n/11		11	9
	Pfeiffer's Functional Assessment Questionnaire (FAQ), n/8		5	3
	Visual Analog Scale – Pain Intensity (VAS)		10	0

Besides the therapeutic effects of tDCS, ameliorating one or more symptoms related to mild neurocognitive disorder, depression and chronic pain, our protocol also improved several functions, including executive and attention functions (Digit Span), verbal fluency (Letters – FAS), comprehension and reasoning (Similarities – WAIS), episodic recall (RAVLT), nonverbal recall (ROCFT) which were already relatively well preserved in this elderly patient before the first tDCS clinical trial.

## DISCUSSION

In line with our results, tDCS over the DLPFC has been proposed as a promising tool for restoring cognitive function in the context of memory decline related to both mild and severe neurocognitive disorder.^3^ Early interventions using this novel tool in the preclinical phase of Alzheimer's disease (AD) might be potentially disease-modifying, promoting neuroplasticity, and may result in neurocognitive enhancement in mild neurocognitive disorder. tDCS has shown improvements in episodic memory,^14^ suggesting that anodal left DLPFC tDCS might strengthen existing episodic memories and reduce memory loss in older adults with MCI.^3^

The potential for tDCS to increase learning and cognition may well lead to the development of enhanced therapeutic interventions. For example, previously reported studies have also shown therapeutic effects of tDCS in executive functions with anodal tDCS over the left DLPFC,^9^^,^ 11 ^,^ 14 ^,^ 17 and possibly reflects neuromodulatory effects on the left cerebral hemisphere.

The antidepressant-like effects observed were probably due to tDCS treatment since cognitive training (CT) is not intended to treat depression, although it may have some effect on some aspects, for example on ruminant thoughts. A number of studies have linked depression with reduced activity in the left DLPFC, which may account for the negative emotional bias, that is, the tendency of depressed individuals to show enhanced attention to and preferential memory for negative information. Although there are suggestions of an important synergy of these two therapeutic modalities,^24^^,^ 25 this seems unlikely in our study considering that CT was combined with tDCS only during the first tDCS course.

tDCS has also been used to alleviate chronic pain.^6^^,^ 7 Our second protocol included M1 stimulation and was chosen in view of the patient's complaint of sciatic nerve pain. Many studies have shown that M1 anodal tDCS has an analgesic effect. Our results demonstrated that the patient experienced improved quality of life not only because of improved cognition but also as a result of decreased pain perception after tDCS treatment. A similar profile of tDCS in alleviating pain has been previously reported.^26^

In conclusion, this report illustrates potential benefits of anodal tDCS sessions over the left DLPFC in a 78-year-old patient with mild neurocognitive disorder and complaints of depression and chronic pain. Further controlled studies involving a larger number of patients are required to ascertain the potential benefits of this non-invasive technique in mild neurocognitive disorder cases.
